# Adulteration detection technologies used for halal/kosher food products: an overview

**DOI:** 10.1007/s44187-022-00015-7

**Published:** 2022-04-20

**Authors:** Mustafa Mortas, Nour Awad, Huseyin Ayvaz

**Affiliations:** 1 Department Food Engineering, Faculty of Engineering, Ondokuz Mayıs University, Samsun, 55139 Turkey; 2 Department of Food Science and Technology, The Ohio State University, 110 Parker Food Science and Technology Building, 2015 Fyffe Road, Columbus, OH 43210 USA; 3 Department of Food Engineering, Faculty of Engineering, Canakkale Onsekiz Mart University, Canakkale, 17100 Turkey

**Keywords:** Halal, Kosher, Adulteration, Authentication, Detection techniques and methods

## Abstract

In the Islamic and Jewish religions, there are various restrictions that should be followed in order for food products to be acceptable. Some food items like pork or dog meat are banned to be consumed by the followers of the mentioned religions. However, illegally, some food producers in various countries use either the meat or the fat of the banned animals during food production without being mentioned in the label on the final products, and this considers as food adulteration. Nowadays, halal or kosher labeled food products have a high economic value, therefore deceiving the consumers by producing adulterated food is an illegal business that could make large gains. On the other hand, there is an insistent need from the consumers for getting reliable products that comply with their conditions. One of the main challenges is that the detection of food adulteration and the presence of any of the banned ingredients is usually unnoticeable and cannot be determined by the naked eye. As a result, scientists strove to develop very sensitive and precise analytical techniques. The most widely utilized techniques for the detection and determination of halal/kosher food adulterations can be listed as High-Pressure Liquid Chromatography (HPLC), Capillary Electrophoresis (CE), Gas Chromatography (GC), Electronic Nose (EN), Polymerase Chain Reaction (PCR), Enzyme-linked Immuno Sorbent Assay (ELISA), Differential Scanning Calorimetry (DSC), Nuclear Magnetic Resonance (NMR), Near-infrared (NIR) Spectroscopy, Laser-induced Breakdown Spectroscopy (LIBS), Fluorescent Light Spectroscopy, Fourier Transform Infrared (FTIR) Spectroscopy and Raman Spectroscopy (RS). All of the above-mentioned techniques were evaluated in terms of their detection capabilities, equipment and analysis costs, accuracy, mobility, and needed sample volume. As a result, the main purposes of the present review are to identify the most often used detection approaches and to get a better knowledge of the existing halal/kosher detection methods from a literature perspective.

## Introduction

The disclosure of eating habits is a complex matter, and there are many factors that could explain food choices and preferences [1]. These factors can be divided into intrinsic factors (appearance, taste, quality, and presentation methods of food), extrinsic factors (adverting, seasonal variations), socioeconomic factors (family income, food costs), biological factors (age and gender of consumers), personal factors (level of expectations, the influence of other people), cultural, religious and regional factors [2]. Among these factors, religion is a vulnerable for food production for which there are some special regulations.

Although there are no food prohibitions in Christianity, some prohibitions and/or aspects of ritual slaughter exist in Judaism, Islam, Hinduism, and Buddhism [3, 4]. Halal slaughter requires that carcasses be bled to optimize blood loss. This religious procedure purifies the animal, making it suitable for human consumption. The halal slaughter procedures of different countries are consistent with halal regulations in terms of blood loss. In addition, the religious slaughter of mammals and birds in Judaism must be in accordance with Jewish dietary regulations. It must be a kosher animal (ruminants with split hooves that masticate their cud, or domestic birds that are not birds of prey). The animal is slaughtered by cutting its neck (below the glottis) with an extremely sharp, nick-free knife and allowing the blood to flow out [4]. According to Manusmriti, a Hindu law code, the slaughter of animals, except for ceremonies or for consumption, was strictly forbidden.The code spoke of sacramental sacrifices of certain animals and the consumption of their flesh under certain conditions. Jhatka is a method of killing an animal according to Hindu law to obtain meat for Hindu consumption. Simply, the method is based on decapitating the animal with a single blow to the nape of the backbone to cause instant death and give the animal no opportunity to feel or recognize pain. The jhatka procedure is performed exclusively on goats and sheep (Sikhs and Hindus believe that cattle are sacred; therefore, meat consumption is prohibited [4].

Fraud can be broadly described as "the intentional deception for personal gain or to harm another person." Food fraud control is mainly concerned with determining the amount of a marker compound and comparing it with the values of the test material [5]. Various types of food fraud can be observed, such as mislabeling,unapproved additives, and inedible meat from animal feed stores. The inedible meat was produced in different ways: freezingcaused by poor transportation and storage conditions, unclean or diseased, unlicensed, and unsanitary meat [6].

Adulteration and authenticity are two important criteria for food quality [7]. Food fraud has existed a long time since food has been offered for sale. The first adulteration method was replaced sugar with chalk in baked goods [8]. To date, various methods of adulteration have been used ranging from adding natural substances such as sugar to improve according to consumer choice, to obtaining financial benefits by mixing cheap and low-quality honey with honey to increase yield [9],to very harmful compounds such as melamine added to both milk and wheat gluten to fraudulently improve apparent protein content. In China, numerous health problems are reported in infants and young children from the consumption of melamine-contaminated infant formula and related dairy products [10]. Adulteration of meat and meat products was not a significant problem for a long time because the meat was consumed fresh or processed to a small extent, but with the improvement of technology and storage facilities for meat adulteration became a major problem. However, meat adulteration methods are based on the use of low-quality meat instead of high-quality [11], and the consumers are unable to detect adulteration, especially in highly processed meat products [12]. Food fraud has a large economic potential, believed to be worth several billion dollars every year. For example, it was reported in Guardian newspaper, that 900 people were jailed in China for meat fraud involving 20.000 tons of unsuitable meat including mink, rat and fox. In addition, 4o% of lamb meat was sold for takeaway dishes that contained other types of meat.’[13].

Lard is one of the cheapest fats in the food industry. It can be easily combined with other fats to reduce production costs. In general, lard gains its importance and controversy from two perspectives: (1) economic considerations, (2) religious restrictions [14]. Lard consists of trans-fat, which is preferred in the food industry due to its ability to solidify and stabilize vegetable oils and extend the shelf life of food. On the other hand, it has many disadvantages from a medical point of view, because it is known that a diet high in lard contributes to hypertension, colon, breast, and prostate cancer [14]. The fats in lard are mainly present as triglycerides and are distributed between saturated and unsaturated fatty acids. While most animal fats and vegetable oils are composed of the same chemical components, they differ in structure. Accordingly, identification and differentiation of lard impurities difficult due to subtle differences in fatty acid composition [15].

The terms 'Kosher' and 'Halal' are religious food regulations for 'Jews' and 'Muslims,' and these terms are in vogue and essential for producing suitable food products that meet religious requirements There are several factors to consider when evaluating whether a food product is halal or kosher. (1) Muslims and Jews must ensure that all foods do not contain pork or other unacceptable animal byproducts such as blood or other ingredients. All foods containing pork [16] or its derivatives, such as ham, bacon, and sausage are prohibited for the Muslim and Jewish communities [17]. However, while moderate and general consumption of alcoholic beverages is permitted in Judaism, it is strictly forbidden in Islam. (2) Method of Slaughter. In the Islamic method, the slaughtering process should be carried out on a special area of the animal’s neck with a sharp knife [4]. Based on what has been said, food fraud is a very important issue nowadaysIn order to meet the expectations of consumers, the authorities have established some special regulations and conditions for the food industry. In addition to the production of suitable food products, the fast and accurate detection of foods products that are unsuitable in terms of halal-kosher status is another important critical issue. Accordingly, the scope of the current review is to outline the analytical methods used in monitoring the halal/kosher status of foods and to examine the superiority and inferiority of the techniques discussed. Many methods, including different types of instruments, have been improved for this purpose.

### Religious aspects of meat and animal-based foods

Religion influences people’s behavior, well-being, and lifestyle [18], and it is a kind of necessary cultural asset that controls and influences consumers’ purchasing decisions [19–21]. Nowadays, with the growing capacity of halal and kosher production in the food industry, there are restrictions not only on different types of meat, but also on pig derivatives such as gelatin and/or lard in meat products, as in the cases of sausage, meatballs, mixed animal fats [16, 22, 23], cakes [7], chocolate products [24] butter [25] and cookies [26]. Given this wide range of foods susceptible to fraud, consumers should be more familiar with halal/kosher terminology to improve their understanding and knowledge of food options and restrictions.

### Halal

'Halal,' is an Arabic word meaning ‘lawful’ or ‘permitted’. the Aspects of Muslim food choices depend on lifestyle, economics, culture, diet, health concerns, and halal status, which includes dietary laws from the Holy Quran [27]. Food must have some characteristics to be halal according to the laws. These include prohibitions such as not eating foods and beverages containing alcohol, pork, and pork by-products; blood and blood-based products; improperly slaughtered animals, and eating dead animal meat.

Meat and animal-based foods are a significant topic of discussion in the halal food issue. The global Muslim community, which accounts for 1.8 billion people or 23% of the world's population, is nowadays driving demand for halal foods that are in line with their religious beliefs. As a result, the halal industry is the fastest-growing market in the world accounting for, one-third of the global market [28]. Halal products have gained global acceptance, and the value of the halal food industry was $ 587.2 billion in 2004 and grew to $ 641.5 billion in 2010 [29].

More recently, during the new COVID-19 crisis, the value of the global halal food industry was about 1.7 trillion USD in 2020, and it is estimated to reach a size of 3.2 trillion $ by 2027 [30]. Halal food production is expected to exceed 20% of world trade in line with the expectation that Muslims will account for 30% of the world's population by the end of 2025 [17]. In response to this growing demand, food manufacturers have tended to acquire halal certificates and increase their halal production. On the other hand, this trend also increased the number of manufacturers who produced adulterated food products.

### Kosher

The term 'kosher' can be defined as 'fit and proper. This term refers to religious law that contains Jewish dietary principles described in the Torah, the most important text of Judaism, which contains the 613 commandments of the Five Books of Moses. Similar to halal, the term "kosher" also influences the food preferences of some consumers. Many food manufacturers align their productions with consumer demands for valid kosher practices, where kashru (Jewish dietary laws) covers three areas, namely the selection and slaughter of animal foods, the koshering of meat and poultry products, and the separation of meat and dairy products [31]. As mentioned earlier, meat should have some characteristics to be kosher. For example, it should come from a kosher animal, have a slaughter acceptable to kosher animals (as defined by the Torah) and not contain blood or tissue containing blood. Hence, kosher animals may be listed as follows: (for bovine and ovine) ruminants and animal with cloven hooves as cow, goat, deer, and sheep, (for poultry) chicken, turkey, goose, pheasant, and duck [31]. However, since pork and its derivatives are not considered kosher foods, their inclusion in animal foods is considered food adulteration. Statistically, kosher food sales accounted for $24 billion of the total global food market, in 2017 and are estimated to increase by 11.5% by 2025 (reported Supermarket News). More recently, during the COVID-19 crisis, it is estimated that the global market for kosher products will reach about $ 26.7 billion and is expected to reach a market size of $ 35.3 billion by the end of (2027) [30]. There are now 11,400 companies producing kosher products around the world. Surprisingly, data showed that 80 percent of kosher consumers in the United States are not Jewish, and they do not consume it for religious reasons. According to the Food Institute Report, 38% of Americans buy kosher foods because of vegetarian choices. About16% of Americans buy kosher foods because they need halal, which is kosher-compliant. People seek kosher goods of nutritional quality (62%),overall health(51%), and food safety (34%)[32].

As the demand and consumption of kosher products increase worldwide, the number of factories and restaurants producing kosher products has increased in recent years [33].

## Detection methods for authentication and adulteration of halal/kosher foods

Meanwhile many methods and techniques have been developed to prove the authentication and adulteration of halal/kosher foods. Authentication of halal and kosher foods cannot be achieved through physical inspection. Therefore, it is essential to use the latest technological, and analytical tools to obtain accurate results [15]. The most effective techniques are 1. Chromatographic Methods (High-Pressure Liquid Chromatography (HPLC), Gas Chromatography (GC), 2. Conventional methods such as Capillary Electrophoresis (CE) and Electronic Nose (EN) Polymerase Chain Reaction (PCR), Enzyme-linked ImmunoSorbent Assay (ELISA), Differential Scanning Calorimetry (DSC), 3. Spectroscopic methods such as Nuclear Magnetic Resonance (NMR), Fourier Transform Infrared Spectroscopy (FTIR), Fluorescence Light Spectroscopy, Near-Infrared (NIR) Spectroscopy, Laser-Induced Breakdown Spectroscopy (LIBS), and Raman Spectroscopy (RS), all of which will are discussed in more detail in the following sections.

### Chromatographic methods

Chromatographic methods are commonly used for the detection of food adulterants. The ability of these methods to detect even minute amounts of any food is the explanation for their widespread use, ranging from edible oil adulteration [34] to meat adulteration [35].

#### HPLC

The detection principle of HPLC is based on the separation of the analyte (sample) from the eluent (mobile phase) with a column (stationary phase). The separation is performed while maintaining the molecular structure of the sample. Qualitative detection of protein profiles of meat by liquid chromatography (LC) methods is one of the applications in food science for the discrimination of meat from a variety of animal species [35]. Meat source identification methods for HPLC are used to detect peptides, protein profiles, and amino acid profiles [36]. Some HPLC-based detection methods for animal species-specific histidine dipeptides, balenine, carnosine, and anserine for separation of animal material mixtures [37]. The detection and quantification of triacylglycerol (TAG) analysis by HPLC has been used as a detection method for meat species [38]. Likewise, TAG analysis has been utilized for the detection of lard in some vegetable oils and animal fats [39]. TAG profiles (fatty acids in the 2-position of (TAGs)) identified by HPLC may indicate adulteration of lard in beef tallow [26]. Rohman et al. (2012) use the HPLC method to classify and differentiate lard and other animal fats (chicken, mutton, and beef) based on TAG composition [40]. Generally, TAGs are known to play a very important role in the differentiation of meat species [41]. Saturated fatty acids in pork fat contain TAG at the Sn-2 position, and this position differs from other meat types. In a study to detect animal fat adulterants in vegetable oils by HPLC, different types of vegetable oils were used, such as palm oil (PO), palm kernel oil (PKO), and canola oil (CLO). They were blended with different proportions of animal fats such as lard (GLD), beef tallow (BT), and chicken fat (CF), ranging from 2 to 20%., For each oil 15 mixtures were prepared: 98:2, 95:5, 90:10, 85:15, and 80:20 (w/w), characterized by the mass ratio of vegetable oil (VO) to animal fat (AF) (VO: AF). The main purpose was to study the changes in the composition of triacylglycerol (TAG) in the oil samples by (HPLC) before and after adulteration. The results showed that visual comparison of TAG profiles of contaminated PKO can be used to determine the presence of lard impurities in PKO. However, this method was ineffective for PO and CLO [42]. The literature supports the effectiveness of both gas chromatography (GC) and high-performance liquid chromatography (HPLC) as useful tools for detecting lard contamination in fried products. Data also showed that Palmitic acid enrichment factor (PAEF) parameters calculated from GC analysis of fatty acid methyl esters and triglycerol profiles (TG) from HPLC can evaluate fried products. The results suggested the efficiency of PAEF for investigating for four fried products (peanuts, tempeh-fermented soybean cake-, chicken, and beef), as well as TG, can be used to detect lard in fried chicken and tempeh products [42]. Protein, on the other hand, is another indicator of meat adulteration. In published research, HPLC combined with electrochemical (EC) detection technique was developed to distinguish fifteen meat products from fifteen different animal species. The number of peaks and peak retention time may easily distinguish meat varieties from each other. As the chromatographic profile showed two-peak for horse and scallop, three-peak for deer, chicken, ostrich, cod, crab, salmon, shrimp, and bullfrog, and four peak patterns for beef, goat, pig, and duck. In the same study, they claimed that by comparing peak features (area, retention duration, etc.), they could readily differentiate a mix at 1:1 ratio of beef, pig, and horse. Two peaks at 7.6 and 12.1 min were obtained at greater detection sensitivity (2 V full-scale level), indicating the presence of horse meat in the mixture. There were no interfering peaks from beef or pork at this sensitivity [35].

In addition, Hoffmann et al. (2016), used the High-Performance Liquid Chromatography-Tandem Mass Spectrometry (HPLC–MS/MS) for sensitive detection of lupine (*Lupinus angustifolius*), pea (*Pisum sativum*), and soybean (*Glycine* maxima) in meat products [43]. The discrimination of pork from beef, mutton, chevon meats, and chicken depends mainly on their primary amino acid content and can be performed by reversed-phase high performance liquid chromatography (RP-HPLC) with O-phthalaldehyde (OPA) and ultraviolet (UV) detection. The most distinguishable amino acids between pork and other meats were arginine, alanine, histidine, serine, and valine [44]. TG, TAG, proteins, and amino acid detections are used for meat adulteration by HPLC systems, but the analyzes for HPLC system require difficult extraction methods and long time [35]. However, mass spectrometry (MS) can be combined with HPLC system, but it is associated with high cost and many signals for meat differentiation. In a study, the HPLC–MS/MS based detection method was used for processed horse meat and pork and it was found that the identified marker peptides were stable, as well as the method was fast and sensitive at 0.24% in the matrix system [36].

#### Gas chromatography (GC)

Another chromatographic method used in meat adulteration is Gas Chromatography (GC). In analytical chemistry, GC is a common method of chromatography for separating and analyzing compounds that can be vaporized without decomposition. GC is often used to determine the purity of a product or to distinguish the different components of a mixture. It can be used to detect oil and fat-based foods in terms of fatty acid composition. In addition, fatty acid composition is a specific indicator for the detecting adulteration. The C20:2 marker is used for fatty acid analysis by GC for detection of pork in processed meat products. The detection limit pork has been reported to be 2% [45]. Another factor used for adulteration detection by GC is a volatile compound collected by solid-phase micro extraction (SPME) and analyzed by gas chromatography and mass spectrometry (GC–MS) [46]. Volatile compounds in pork can be detected by SPME in combination with GC for halal/kosher status. In a study by Estevez et al., volatile compounds were investigated in different meats (raw, chilled, cooked, and chilled cooked) from Iberian and lean pigs [47]. The 57 preliminarily identified volatile were classified into 11 classes, and the volatile compound profiles of both types of cooked pork were quite similar, but showed quantitative differences. Lipid derived volatiles such as acids (nonanoic acid), ketones (octane-2,3-dione), aldehydes (pentanal, hexanal, heptanal, octanal, nonanal, decanal, het-(E)-2-enal, dec-(E)-2-enal, deca-(E, Z)-2,4-denial, and undec-(E)-2-enal), furans (2-pentyl-furan), and alcohols (hexane-1-ol, oct-1-en-3-ol, and octan-1-ol) were detected as most abundant compounds [47].

Hexanal (dominant aldehyde) was detected in cooked meat of both groups, and limits for hexanal and non anal compounds were 58 ppb and 13 ppb, respectively. Trivedi et al. published a study to detect the adulteration of beef and pork using GC–MS and UHPLC-MS. GC–MS was chosen because it allows the study of metabolites involved in primary metabolism [48]. Further, GC can be used in combination with SPME to detect volatile compounds in pork (cooked, frozen, raw), to differentiate various types of pork have different volatile profiles due to enzymatic and oxidative deterioration, fat content, and compositional characteristics[47]. Different meats and sausages (beef, mouflon, chicken) have been distinguished from pork and sausage using EN and GCMS-HS (Headspace Analyzer) [49]. However, sample preparation prior to GC analysis is very lengthy and demanding. For example, in the detection of fatty acid composition, methylation steps must be performed to convert fatty acids to fatty acid methyl esters [50]. In addition, laboratory equipment and chemicals are needed for various purposes such as mobile phase, drying, and vacuum.

### Conventional techniques used to determine halal/kosher food products

#### Mass spectroscopy based approaches

Mass spectroscopy-based approaches for food substances are powerful analytical techniques with excellent capabilities [51]. These techniques can detect analytes (ions) as mass-to-charge (m/z) ratio. The MS technique has contributed to the improvement of food quality because of its ability to establish between food characteristics and overall food quality. Nowadays, one of the most analytical techniques for the identification of contaminants in food is based on MS detection, since this method allows the quantification of substances with great selectivity and sensitivity. The literature describes effectiveness of the MS technique in the detection of chemical contaminants (drug and pesticide residues), and microbial contaminants (pathogens and toxins)[52]. For the detection of the authenticity of halal and kosher products, there are only a few published works that need further investigation. For example, orthogonal projection onto latent structures (OPLS-DA) using GC/MS data has shown good sensitivity for the detection of 5% of lard and beef tallow in canola oil [53].

The GCMS-HS technique was used to identify a total of 43 volatile components of pork and other sheep, cow, and chicken products [49]. The identification and differentiation of fat species such as lard, chicken, beef and mutton was performed using Elemental Analyzer-Isotope Ratio Mass Spectrometry (EA-IRMS) [54]. In another recent study by Sairin et al., gas chromatography-mass spectrometry (GC–MS) was used to detect lard adulteration by the classifying fats from different animal sources [55]. In the aforementioned study, the classification of different animal fats was based on their dielectric spectra. The data showed that lard had the highest dielectric constant spectra among other animal fats and was mainly influenced by the composition of C16 and C18 fatty acids [55]. A recent study by Heidari et al. addressedthe identification of adulteration of olive oil with other cheap oils, including lard, by using gas chromatography-mass spectrometry [56]. This approach, is simply based on measuring the changes in the profile of fatty acid methyl esters (FAMEs) of the oil profile. The results proved that this technique is able to detect lard adulteration even with a low percentage of lard (5%w/w), with an error of less than 2% [53].

#### Capillary electrophoresis (CE)

One of the electrokinetic based separation methods with some factors such as molecul size,distribution etc. is Capillary Electrophoresis (CE) [57]. The capillary electrophoresis (CE) method is similar to HPLC in terms of its ability to provide rapid and accurate quantitative results. It is therefore thought to be an electrophoretic analog of HPLC [58]. CE methods have some applications for food analysis and also food authentication [59]. It has been used for authentication of meat with egg-white [60], soybean, lupin and pea [61]. In generally, CE is a sufficiently sensitive protein analysis technique but not so sensitive that it is commonly used to detect meat adulteration [62]. Inauthentic meat may involve the use of low-quality meat instead of high quality meat, the addition of extra connective tissue or fat in excess ofthe normal amount, and the addition of water, non-meat proteins or other substances [58]. Linear discriminant analysis with CE-SDS was used to differentiate and identify different types of meat [63]. Likewise, a polymer capillary gel electrophoresis (SDS) polymer-filled capillary gel electrophoresis (CE-SDS) has been used for the determination of meat proteins to achieve species differentiation. In the research, sarcoplasmic and myofibrillar proteins were extracted from raw bovine, turkey, and porcine muscles and prepared for CE-SDS analysis. Using the optimized CE-SDS method, sarcoplasmic protein profiles were specific for each species. Both qualitative and quantitative separations could be performed to differentiate and identify the authenticity of the meat species. Exchangeable polymer-filled capillary electrophoresis (SDS-CE) combined with linear discriminant analysis (LDA) is a good pattern for protein profiles for meat species identification. This technique can be used for halal/kosher identification and evaluation of meat species. In published research, capillary electrophoresis-sodium dodecyl sulfate polymer-filled capillary gel electrophoresis (CE-SDS) has been used for meat species identification (beef, turkey, pork, and turkey). They collect quantitative data on water-soluble and salt soluble proteins of different meat species. The correct categorization offered by linear discriminant analysis (LDA) for water-soluble protein data was reported to be 100 percent for every meat type except pork (94%). In contrast, for salt-soluble protein data, the correct classification was 88% for beef and mechanically deboned turkey, and 94% and 100% for turkey and pork, respectively [63].

In another study by Vallejo-Cordoba, (2005), it was shown that the sarcoplasmic and myofibrillar proteins (water soluble extracts) can be used to differentiate and identify meat species (beef, pork, and turkey) using the CE-SDS method [58]. Accordingly, the presence of myosin, the most abundant of the salt soluble proteins, was observed and identified using a broad molecular weight standard. Anionic and cationic water-soluble polydisperse polyelectrolytes can be used for the separation of sarcoplasmic proteins from chicken and raw beef. The reagents have reduced protein-wall interaction and improved resolution of muscle extracts' due to ion exchange/ionpairing interactions between analyte and polymer. In general, the CE identification method has some advantages and disadvantages because it provides high-resolution separations at minimal cost in terms of sample size, reagent consumption, and operator time [57], high efficiency, high throughput, and high speed [59]. On the other hand, the sample must be pretreated chemicals for analysis. Besides, the presence of an expert person is a very important factor for improving separation capabilities and analyzing the results.

#### Electronic nose (EN)

The ‘electronic nose' (e-nose) is a technical device based on copying human sense of smell [64] that is mimicking the work of the human sense of smell [65]. It is used for food aroma analysis, generally without separation and identification of volatile compounds. A typical e-nose mainly consists of three parts: (1) the sampling system, (2) a set of non-selective sensors or a mass spectrometer (MS), and 3) a system for recognizing products by their specific odors [66].

The standard e-nose equipment is based on a series of gas sensors capable of detecting the chemical signal from the headspace and transmitting it to the electronic devices. Different types of sensors can be used for different applications, including electrochemical sensors such as metal oxide semiconductors (MOS) or conducting polymers (CP) and piezoelectric sensors like quartz microbalances (QCM) [65]. The applications of EN vary widely, ranging from medical applications [67] to environmental monitoring [68], food spoilage detection [69] and from pharmaceutical applications [67] to chemical sensor arra development [70].

There are many applications of the e-nose technology in food analysis [71]. E-nose can be used for measure the freshness of fish [72], determine meat quality, identify spoilage, and detect off-flavors [71]. Besides, e-nose can not only determine the quality of meats, but also address the adulteration [73], as well as the analysis of volatile compounds, especially aldehydes of cooked pork [74]. Accordingly, e-nose can be used to detect pork and its derivatives for halal/kosher authentication. In one study, an electronic nose (zNoseTM) that senses surface acoustic waves (SAW)was used to detect lard in refined, bleached, deodorized (RBD) palm oil spiked with lard at levels ranging from 1–20% (w/w). Detection of lard adulteration could be determined from a few distinct specific peaks in the chromatogram, and lard adulteration of RBD palm oil could be detected up to 1% using zNoseTM [75]. Inaccording to another published study, four different meat samples (sheep, cow, chicken, pork) and three types of sausages (pork, chicken, beef) were analyzed using a gas chromatography-mass spectrometer with headspace analyzer (GCMS-HS) and a surface acoustic wave (SAW) detector with the electronic nose [49]. They found 43 major volatile compounds in pork by GCMS-HS and seven compounds in raw pork, and 13 compounds in pork sausage detected by electronic nose. The result showed that electronic nose is a rapid, accurate, cost effective, and environmentally friendly tool for the detection of porcine-based ingredients in foods, which is particularly important for the halal authenticity and verification of halal [49]. An e-nose method has been used for predict the degree of pork in minced mutton using with metal oxide sensors as described by [76]. Latief et al. used the e-nose technique for rapidly identify lard adulteration in three different liquid forms of lard, beef, and chicken. The data showed the ability to identify lard with the highest accuracy of nearly 95.6% [77]. However, the change in volatile compounds of meat may indicate deterioration (enzymatic, chemical, and microbial) during cold storage [78]. There are so many variables that affect the volatile compounds of meat, such as fatty acid compositions[79], fat content [47], meat types, microbiological activity [80], and cooking and storage temperature. A e-nose method for detection of ethanol content at different types of alcoholic drinks was developed by Ab Mutalib et al. [81].The have developed an electronic e-nose for detecting ethanol content in different types of alcoholic beverages Moreover, the use of electronic nose has some advantages, such as the fact that no sample pretreatment or chemicals are required for the analysis [82]. On the other hand, the threshold value may be affected by various factors. In addition, the detection range of e-nose is limited to volatile compounds. However, literature suggests a wide use of this technique in the future due to the lack of effective application of this method to achieve its optimal efficiency Table 1.

#### Polymerase Chain Reaction (PCR)

Polymerase Chain Reaction (PCR) detection is based on the hybridization of specific oligonucleotides (a target DNA) and their synthesis [83]. The DNA-based methods for meat species identification are widely used for identification analysis [38]. Some methods for meat authentication rely on lipid or proteinsidentification. However, DNA-based methods can be used for highly processed and heated meat products because they are more thermally stable than many proteins [84]. Proteins are heat sensitive substances, and therefore degrade during food processing. PCR is one of the DNA-based specific and sensitive techniques for detecting components of animal origin in food [85], and detection of halal and kosher authentication with PCR is possible [84]. There are several PCR techniques for differentiation of meat species, and they can be listed as follows: simplest agarose gel electrophoresis for fragment size verification method DNA fragment amplification [83], sequencing of DNA amplification (PCR-sequencing) [86], PCR single strand conformation polymorphism (PCRSSCP) analysis [87], mitochondrial cyto- chrome *b *gene [88] simultaneous amplification of two or more fragments with different primer pairs (multiplex PCR) [89], duplex PCR–Enzyme-Linked Oligonucleotide Assay (ELONA) [89] analysis of PCR-restriction fragment length polymorphism (PCR–RFLP) [90], Park et al., analysis of randomly amplified polymorphic DNA (PCR-RAPD) [91], real-time fluorescent PCR assays [92], and short sequence repeat markers (microsatellites) or DNA chips (microarrays) [93]. For meat authentication, some markers used in DNA methods can be listed; mitochondrial genes [94], cytochrome b [95], the 12S and 16S ribosomal RNA subunits [96], displacement loop region (D-loop) [97], growth hormone gene [98]. Some studies on the detection animal species by PCR have shown that this technique is highly specific, sensitive, and useful compared to protein-based methods [99]. There are several published studies on the PCR-based detection method of pork, horse, and other unsuitable meats for halal/kosher authentication. In research, PCR-restriction fragment length polymorphism (RFLP) has been used to differentiate beef, pork, buffalo, quail*,* chicken*,* goat*, and* rabbit species for halal authentication. PCR products were obtained from the *cyt b* gene of these samples. The detection was successfully achieved by genetic differences within the *cyt b* gene between meat samples [73]. Similarly for the detection of porcine or wild boar [90, 98] horse meat [90, 100].

PCR is also used for the detection of horse, cat, a real-time PCR technique for the detection of pork and horse meat [92–102], porcine and bovine gelatin mixtures and capsules [103]; primer multiplex PCR (CP-M-PCR) method for the detection of pork and horse [104]. The PCR method combined species-specific primers with the genetic marker (Cytochrome b, D-loop region) for pork meat [89], as it could detect even 1% pork in raw beef or chicken mixtures using the duplex PCR–Enzyme-Linked Oligonucleotide Assay. The real-time PCR-based fluorescence technology has several advantages: it is a rapid method without extensive preparation (sequencing, enzyme digestion, or conformational analysis), high-throughput screening for multiple samples, and avoidance of electrophoresis required by other PCR methods [105]. The presence of porcine derivatives in various foods was investigated using genomic DNA. In addition good and bad genomic DNA was found in foods for sausages and bread/biscuits, respectively [99]. Another study by Rosman et al. (2016) investigated the reason for the deficiencies in the ability of PCR to detect lard adulteration in chocolate. Four basic chocolate ingredients, sugar, milk powder, cocoa butter, and cocoa powder, were adulterated with lard and analyzed using the PCR technique. The results revealed that cocoa powder has an inhibitory effect, which makes the extraction of lard DNA from chocolate adulterated with lard challengi[106]. Consequently, the PCR assay has been shown in the literature to provide excellent results in detecting pork adulteration in food and is a potentially reliable technique for halal authentication [99]. However, the PCR technique requires high equipment and operating costs, as well as complex and sensitive sample preparation [83].

#### Enzyme-linked immunosorbent assay (ELISA)

Enzyme-linked immunosorbent assay (ELISA) is another technique for food authenticity detection. ELISA is a biochemical technique mainly used in immunology to detect an antibody or antigen in a sample [107]. Therefore, ELISA enables highly sensitive and selective quantitative/qualitative analysis of antigens, including proteins, peptides, nucleic acids, hormones, herbicides, and secondary plant metabolites [108]. However, for the detection of meat species, various test kits have been produced by some companies for the identification of meat species in cooked, raw, and processed meat and meat products [109]. ELISA tests using food constituent analysis can be divided into two methods the the sandwich and indirect ELISA [110]. In the indirect ELISA method, two antibodies are used to bind to a specific antigen [111]. In ELISA detection, specific antibodies must be used to accurately detect the meat species (Hsieh et al., 1997). There are two types of specific antibodies, namely polyclonal and monoclonal (MAbs) [112]. The use of MAbs has several advantages: It reduces the cost and improves the quality of data from the test [113]. According to several studies, the ELISA method has been used to detect soy components in some meat products such as sausage, hamburger, etc. [114–116] and used for qualitative and quantitative discrimination of meat species. For example, a monoclonal antibody against a thermostable porcine muscle protein was used to detect pork in cooked meat products. The method is capable of detecting skeletal muscle (except cardiac muscle), smooth muscle, blood, and non-muscular organs of pork. In heterologous meat mixtures, the detection limit was set at 0,5% (w/w) pork, and the intra and inter-assay coefficients of variation were 5.8 and 7.9%, respectively [117]. Martin et al. tried to attempted 1–75% raw pork in raw ground beef by ELISA using forensic-grade anti-porcine glycoprotein immunoglobulin. The detection limit and coefficient of variation (CV) of the test were 1% and 18%, respectively. They successfully performed a quantitative evaluation of pork adulteration in raw ground beef using ELISA [118]. Mandli et al. used rapid ELISA and immunosorbent assays using HRP conjugated polyclonal anti-pig IgG antibody to detect low levels ( 0.01%) of pork adulterants in beef [119]. In a research sandwich MAb ELISA test kits were used to determine meat species in meat and meat products (100 samples). A total of 22 out of 100 cooked or raw meat and meat products contained undeclared meat species as beef was determined to be horse and deer meat [112]. Small amounts (2%) of pork in beef or mutton samples (heated at 70, 100, 120 ºC) can be detected by ELISA. The detection limits for the analysis of ophidine dipeptides were 2–5% pork in cooked and processed meat [45]. However, MAbs against porcine thermo-stable muscle protein have been developed and have been used to quantify pork in raw and heat-treated meat and feed [120]. For pork in heterologous meat mixtures, the detection limit was set at 0.5–0.05% (w/w). In this study, a pair of monoclonal antibodies (MAbs 8F10 and 5H9) were used with Mab, 8F10 specific for skeletal muscle troponin I (TnI) and biotin-conjugated MAb 5H9 specific for prok [120]. In a more recent study, Jiang et al. (2020) conducted a study to identify two monoclonal antibodies specific for mammalian skeletal troponins (sTn) (mAbs 6G1 and 8F10) and used them to develop a mAb-based sandwich enzyme-linked immunosorbent assay (ELISA) for mammalian meat detection [121]. In general test kits for the detection of meat species can be divided into two groups: ELISA tests and lateral flow devices. Lateral flow tests have some characteristics like qualitative tests and are faster than ELISA tests. In addition, ELISA tests are used for quantitative analysis, but tests provide rapid, reliable, and cost-effective screening for meat species identification [122, 123]. ELISA technique has many advantages such as (i) simple procedure, (ii) high sensitivity, (iii) high efficiency, (iv) safe and environmentally, (v) cost-effective. On the other hand, it also has some disadvantages (i) antibody preparation is a demanding technique, (ii) high probability of false positive or negative findings due to inadequate blockage of the surface with antigen, (iii) instability of the antibodies because they are temperature sensitive proteins that need to be refrigerated during transportation and storage [119]. In summary the use of ELISA assays for food authenticity is convenient and fast, but the sensitivity of ELISA assays is not equivalent to that of DNA-based methods [83, 124] and also varies considerably in a mixed background of multiple species [120]. Table 1 summarizes the use of various conventional methods for the detection of food adulteration.

#### Differential scanning calorimetry (DSC)

Calorimetric techniques and thermal analysis, differential thermal analysis (DTA), thermogravimetry (TG), differential scanning calorimetry (DSC), thermomechanometry, and adiabatic calorimetry have been used to determine food properties and food ingredients for food safety and quality. DSC can be used to determine physical changes such as the glass transition point in food samples by heating. DSC-based methods allow for simple andrapid analysis; addition, the methods require only a few samples to be collected need little sampling. The use of DSC in adulteration problems of fat-based products and edible fats-oil has been started in the last decades [125]. The work that addressed DSC analysis focused on fats and oils in terms of food authentication. The adulteration of body fat with ghee (clarified butter made from milk) was done by Lambelet et al. in 1980. In the aforementioned work, adulteration of ghee with goat fat was detected above a level of 10% using the DSC method. A further study to detect and determine ghee adulteration with other animal body fats like, pork and buffalo fat, using the DSC technique can be recommended as the first study to use the DSC method for halal/kosher authentication in foods [126]. Detection of pig adulteration in foods with DSC was started by Lambelet [126]. In the research, adding small amounts of pork or buffalo body fat to cow or buffalo ghees was detected by additional peaks in the at melting and crystallization curves. However, the DSC method can be used for the qualitative and quantitative determination of tallow in lard. In addition to the adulteration of ghee, DSC detection of lard has also been used for refined-bleached-deodorized (RBD) palm oil [127]. In the low-temperature region, DSC cooling profiles of both genuine and randomly adulterated (detection limit 1%) RBD palm oil samples exhibit a falsification peak as an indicator of the presence of adulteration in the samples [127]. A study conducted by Guntarti et al. investigated the potential use of DSC in combination with multivariate calibration to verify adulteration of wild boar meat in the production of meatball, and the data supported the effectiveness of DSC in analyzing and detecting adulteration [128]. In cooling thermograms of adulterated RBD palm with enzymatically randomized lard (ERLD) oil and genuine lard (GLD), it shows a shoulder peak at − 43.86 ℃. It can be distinguished from adulteration peaks based on other animal fats. The indicator peak of thermograms was found in an adulteration range of 1–20% of ERLD, and ERLD determination by DSC in RBD palm oil is shown to be a more sensitive technique than HPLC and GC techniques [50]. The thermal characterizations of true lard and randomized lard were investigated for their differences. According to the findings of a research that obtained fairly distinct samples, the thermal transitions of randomized lard had five steps, while the other lard had three steps maximum peaks at various temperatures [129]. In a study by Azir et al., the fatty acid composition, triacylglycerol profiles, and thermal properties of lard in cocoa butter were analyzed. The thermal study by DSC showed a total difference between lard and cocoa butter in their heating characteristics [130]. In another study on the adulteration of canola oil with genuine lard, beef tallow, and chicken fat, cooling, and heating thermograms of DSC were examined. The cooling thermogram was found to be unsuitable for adulteration of canola oil, whereas a heating thermogram was used to detect 8% for its adulteration [127].

Lard can be used in foods not only directly as a substance but also indirectly as an excipient. In a published study, foods (tempeh, beef, chicken, and peanut) fried in lard as a frying medium were examined to determine whether lard transferred into the samples [42]. As a result of cooling and heating, DSC thermograms proved useful to determine lard detection (detection limit 10%, w/w) for fried tempeh, chicken, and beef. In another research, thermophysical properties of lard and some vegetable oils/fats (cocoa butter, avocado butter, palm oil, and meat fat) were compared. It was found that lard and vegetable fats (except cocoa butter) have their cooling and heating curves in both low (< 0 ℃) and high (> 0 ℃) melting ranges. The characteristic feature was not found in cocoa butter because the TAG molecular species co-crystallized and melted in a narrow temperature region [135]. On the other hand, thermal analyzes of lard stearin (LS) and lard olein (LO) were studied [136]. The thermal profiles of LS and LO were different from those of the native sample. This difference was based on both the position and the number of thermal transitions. Although the melting profiles are more complicated due to melting kinetics of TAGs and their transition protocols, the cooling thermograms provide a better definition of the different animal profiles adulterated with lard. A study by Dahimi et al. (2014) concluded the following: based on heating profiles, the differentiation of porcine lard (LD) from beef tallow (BT) and chicken tallow (CF) was very clear even at a low dose of less than 1%, whereas the crystallization profiles of BT, which was adulterated with LD, ranged from 0.1% to 1% and from 2 to 5%, respectively [137].In addition CF, which was adulterated with LD, did not exhibit clear changes in its crystallization profiles. Although for the use of DSC to verify the authenticity of foods is feasible, there are few research reports. This limitation may be due to operational difficulties, laboratory requirements, and practicality for industrial expectations, although the method has some advantages such as small sample size.

### Fingerprinting methods

In recent years, fingerprinting techniques have gained increasing interest due to their advanced analytical capabilities. They can generate enormous amounts of data at once. Meanwhile, fingerprinting techniques can be divided into five different categories: Mass spectrometric (MS) fingerprinting, chromatographic fingerprinting, electrophoretic fingerprinting, spectroscopic fingerprinting, and other fingerprinting [5]. Vibrational spectroscopic methods have advanced properties in terms of quantitative information for each component of a complex mixture of analytes in only one spectrum compared to other detection methods [138]. It can be referred to as 'fingerprint' method because it is used for structural identification of organic compounds based on the fundamental vibrations of a particular functional group [139]. Moreover, it has been used not only for food quality analysis, but also for the investigation of adulteration in various foods such as infant formula [140], olive oil [141], palm oil [22]. FT-NIR and FT-MIR spectroscopy. In addition, the methods have been used to detect lard in cakes [7] and chocolate [75]. Recently, Sugito et al. conducted a study using the fingerprinting technique to detect lard in cooking oil. They used contaminated palm oil with chicken oil and lard. The data showed that oil contaminated with lard had the greatest amount of fatty acids, which accordingly showed the greatest change in polarization angle compared to pure palm oil [142].

#### Fourier transform infrared (FTIR) spectroscopy

FTIR spectroscopy is a method for analyzing fats and oils in their entirety. Many researchers are interested in FTIR spectroscopy because it can provide qualitative and quantitative analysis of food constituents, and attracts special attention when combined with chemometrics. FTIR spectroscopy is based on Beer’s law, in which the absorbance of the target substances is represented as a peak proportional to the concentration of the analyte. Therefore, the achievement of peak intensities (maximum absorbance) varies depending on the sample [15]. The FTIR spectroscopy method is one of the methods for determining food authenticity and adulteration. It can be used for various adulterations including the determination of halal/kosher status of foods. FTIR has been used not for only the technical parameters of pork [143] but also for the classification of pork [141, 142] in terms of halal/kosher status. A group of researchers showed that FTIR spectra at wavenumbers of 1000–1200 cm^−1^ in conjunction with partial least squares and principal component analysis are successfully used for quantification and classification of pork in beef meatballs [16]. For the differentiation of muscles from three independent batches of pigs [144]. Detection of Iberian pork sausage [145]. The discrimination of three meats turkey, chicken, and pork) in fresh and frozen forms was investigated using FT-MIR (mid-infrared spectroscopy) [146]. The results of playing PCA to the spectral data showed that the FT-NIR spectrometer in attenuated total internal reflection mode could distinguish chicken, pork, and turkey ground meat based on their infrared spectra and for each meat type. On the other hand, near-infrared transmission and mid infrared were developed using the ATR method to determine fatty acids (saturated fatty acids-SFA, monounsaturated fatty acids-MUFA, polyunsaturated fatty acids-PUFA, C16:0, C18:0, C18:1, C18:2) in fat extracts from pork loin and breast samples. The correlation coefficients (R2) for MIR (ATR) and NIR ranged from 0.91 to 0.98 and from 0.85 to 0.96, respectively [147]. However, the uses of lard are very diverse, ranging from cosmetics [23] to food [7]. For example, some small companies in developing countries illegally add lard to chocolate and their products because it is cheap and can solidify and stabilize vegetable oil [15]. However, only FTIR [24], or FTIR combined with attenuated total reflectance (ATR) and partial least square (PLS) regression[75] have been used to detect lard in a chocolate formulation. Researchers identified, spectral bands associated with lard, cocoa butter, and their mixtures (between 0 and 15% lard content in cocoa butter), and the coefficient of determination (R2) and standard error (SE) were reported to be 0.9872 and 1.305 respectively. They reported that FTIR spectroscopy has the potential to be a rapid analytical tool forquantitative determination of adulterants, especially lard in chocolate. In addition, FTIR spectroscopy was an efficient and accurate method for detecting lard in cake formulation, and the spectral regions 1117–1097 and 990–950 cm^−1^ were selected for detection. Saturated acyl groups and oleic acyl groups (1117–1097 cm^−1^) and trans double bonds (990–950 cm^−1^) peaks were found as peaks in the spectral regions. The obtained R and standard error (SE) of calibration were 0.9790 and 1.7520, respectively. The combination of ATR with PLS regression allowed to extract relevant information from the MIR spectra of fat from cakes [7]. FTIR spectral data in the frequency ranges 3010–3000, 1220–1095, and 968–965 cm^−1^ were used for the detection of lard in a mixture with mutton body fat (MBF) and 1419–1414, and 968–965 cm^−1^ in a mixture with bovine body fat (CBF) [26]. The R^2^ values for lard blends with CBF and MBF were formed to be 0.9866 and 0.9749, respectively. They reported that FTIR spectroscopy allows easy and rapid detection of lard in mutton and cow body fat. The substitution of pork for meatballs may occur due to economic reasons, such as price differences of meat. Indeed, adulteration can be monitored by attenuated total reflectance (ATR) technique, and Partial least squares (PLS) can be used to quantify the amount of adulterated pork in meatballs [23]. The fingerprint region as 1200–1000 cm^−1^ was selected for quantification of adulterant, and the ratio of peak height (R) at 1117 and 1097 cm^−1^ in beef fat (BF) was much higher than that of pork fat (PF). The coefficient determination (R2) and root mean square error of calibration (RMSEC) were reported to be 0.999 and 0.442, respectively. FTIR spectroscopy can be used for the detection and quantification of pork in a meatball. The MID-FTIR method can be used for the detection and quantification of adulterated ground beef with horse meat, fatty beef trimmings, and textured soy protein and the SIMCA (Soft Independent Modeling Class Analogy) method was combined with MID-FTIR for discrimination of adulterated (2–90%) and unadulterated samples. The percentage of adulteration was determined in the spectral region 1800–900 cm^−1^. The values of R^2^, SEC and SEP, were determined as 0.99, 0.0001–1.278, and 0.001–1.391, respectively [26]. In another research, PLS and principal component analysis (PCA) combined with FTIR were used for the analysis of pork fat (lard) in meatball broth. FTIR spectra were determined in the range 1018–1284 cm^−1^ for lard in meatball broth. The values of, root mean square error of calibration (RMSEC) and the coefficient of determination (R2) values were obtained as 1.34% (v/v) and 0.9975, respectively. For halal verification, FTIR spectroscopy, can be used in combination with chemometrics quantitative analysis and classification of lard in meatball broth [148]. FTIR was used in combination with attenuated total reflectance (ATR) to classify unknown gelatin into species of origin, FTIR spectra in the range of 3290–3280 cm^−1^ and 1660–1200 cm^−1^ [149]. The regions were found to provide information on the classification of gelatin source. They found that the study of second derivative of the FTIR spectrum could classify the gelatin source as porcine or bovine. FTIR spectrometry, combined with multivariate discriminant analysis, is able to distinguish halal and non halal ham sausages from China.

In 2012, Xu et al., obtained the best classification performance for discrimination using PLSDA (partial least squares discriminant analysis) with SNV (standard normal variate) spectra (sensitivity 0.913 and specificity 0.929) and LS-SVM (least squares support vector machine) with second derivative spectra (sensitivity 0.957 and specificity 0.929) in terms of prediction sensitivity and specificity [150]. Recently, the FTIR spectrum technique was used in combination with a partial least squares (PLS) method for the rapid detection of lard in sunflower, canola, coconut, olive, and mustard oils [151]. Moreover, a research aimed to developing an analytical method using FTIR spectrophotometry in combination with chemometrics to analyze lard content in grilled and steamed beef sausage with pork has been reported in the literature [152]. In another study, the FTIR method was applied to analyze lardfrom the extraction of "rambak" crackers, a traditional Indonesian food made from different typesof animal skin. The obtained results showed the rapid and reliability of this advanced technique in the identification and classification of lard [153]. FTIR is a vibrational spectroscopic approach that provides molecular structural information [154], and can be used to detect authenticity and adulteration of oil and fat [155]. This method is a fingerprinting method based on spectroscopic determination and can be used for halal/kosher authenticity. Che Man states that the total analysis of lard content in a food sample takes less than 2 min and requires less than 2 mL of the sample [75]. Accordingly, this detection method is accurate, inexpensive, fast, and does not require chemicals. It is recommended as an environmentally friendly tool for quantitative analysis of halal/kosher authenticity. Table 2 summarizes the application of different fingerprinting methods for the detection of food adulteration.

#### Nuclear magnetic resonance (NMR)

Nuclear Magnetic Resonance (NMR) is one of the most important tools for the analysis of food lipids, oils and fats. It is a fast, accurate, and non-invasive technology, is widely used to determine food quality, especially for fruits and vegetables, meat, and aquatic products. NMR is used in the food industry for many reasons, including quantitative analysis and conformational analysis, identification of nutritional or functional properties, quality issues (taste, type, origin, year, etc.), and for process control [156]. Nowadays, NMR technology is one of the most popular techniques for food classification and identification. In general, NMR can identify (1) origin-related issues;. Masoum et al, used this technique to determine the origin of salmon samples[157]. Also, to evaluate the authenticity and origin of honey [158]. (2) Compositional analysis has been used to quickly and accurately analyze the water, fat, and protein contents of foods without altering the original form of food. (3) Quality related aspects, including physical, chemical and structural properties [156]. MR has different types such as low resolution, high resolution 1H and 13C NMR[156]. Low-resolution NMR has been used for solid fat content, melting point curves of semi-solid fats, and weight of oil in food, while high-resolution 1H and 13C NMR has been used for oils, fats, and food lipids [159]. In addition, NMR is one of the available spectroscopic techniques for qualitative and quantitative determination of food safety and quality [160]. In studies to distinguish beef from horse meat, triglyceride signatures obtained by f60 MHz H NMR spectroscopy were compared. Low -field classical triglyceride spectra, typically a 10 min acquisition time, were obtained from fresh beef (76 extractions) and horse (62 extractions). Bis-allylic, olefinic, and the terminal CH3 peaks were three characterizing signals, and the peaks were used for differences between horse meat and beef [160]. Siciliano et al. studied the determination of the fatty acid chain profile of lipids in pork products (two typical PDO Mediterranean salami) during maturation. They reported that the fatty acid chain profiles of total lipid extracts could be determined by quantitative NMR analysis, and that NMR spectroscopy is an alternative to classical chromatographic methods. Canola oils adulterated with lard and beef tallow were successfully identified using proton magnetic resonance (1H NMR), with a minimum detection limit of 5% [53]. Triacylglycerol (TAG) profiles can be detected not only by HPLC but also by NMR as a markers of for halal/kosher issues.

Nurrulhidayah et al. used a high-resolution NMR spectroscopy method for the detection of lard content in adulterated butter, which has specific features such as a peak in the range of 2.60–2.84 ppm [25]. The feature is based on polyunsaturated fatty acids producing seven signals at δ 2.63 corresponding to the chemical shift of double-allylic methylene protons'. In another similar study, Fadzillah et al. used proton NMR spectroscopy in combination with HPLC to authenticate adulterated butter based on the identification of triacylglycerol composition [25]. The data showed peaks in the range of 2.60–2.84 ppm with particular characteristics. The literature stated that only lard has its unique feature, which would give seven signals in the range of δ 2.63 ppm only for polyunsaturated fatty acids. They stated that the TAG composition of lard has been successfully identified as a chemical marker for halal authentication using high-resolution NMR spectroscopy. Accordingly, NMR technique can be used to evaluate the authenticity of halal and kosher foods, but its application is still very rare and limited and needs to be improved. For example, compared with gas chromatography and liquid chromatograph mass spectrometer this technique is associated with high cost, limited detection cability, and low sensitivity. At which point, to create a wider range of applications for this technique, further research and investigation efforts are needed to improve NMR in food identification.

#### Near-infrared (NIR) spectroscopy

Near-infrared spectroscopy is a technique based on measurement of the absorption of electromagnetic radiation in the wavelength range from 750 to 2500 nm. NIR spectrometers consist of a light source, wavelength selector system, sample detector, optical detector, and data processing/analysis system. These parts should be selected according to the intended use to obtain an effective system [161]. Several studies have reported the capabilities of NIR spectroscopy in meat quality evaluation. There are many applications of NIR spectroscopy for characterization of meat fat and meat products, such as prediction of chemical components (wavelengths above 1100 nm), prediction of technological parameters and sensory properties, prediction of carcass fat quality, prediction of meat product quality, and classification and identification of meat and meat products [161]. Mamani-Linares et al. used NIR spectroscopy to successfully identify cattle, llama, and horse meat from homogenized meat and meat juice samples (89–100% of samples were correctly categorized) [162].

Similarly, NIR spectroscopy was used to detect the presence of pork in veal sausage with a contamination level as low as 10% [163]. This application could be used for halal or kosher verification [161]. Prevolnik et al. also used NIR spectroscopy (400–2500 nm) on homogenized samples to determine the chemical composition of a variety of raw meat and meat products, including a variety of pork muscles and muscles from several species [161]. Ding and Xu used Vis–NIR spectroscopy (400–2500 nm) to check pork, mutton, skim milk, or wheat flour in raw and cooked beef patties. The classification rate of the raw and cooked samples were 83.5 percent and 81.6 percent, respectively. The coefficients of determination (R2) for pork, mutton, skim milk powder, and wheat flour were 87, 84, 98, and 99 percent, respectively, using partial least squares regression (PLSR) [164]. However, Vis–NIR spectroscopy has been used to detect numerous adulterants in fresh and thawed ground beef (400–2500 nm) [165]. The adulterants tested included pork, beef fat, and beef offal (kidney, liver, heart, and lungs). The Partial Least-Squares Regression (PLSR) method was used to predict these adulterants, and R^2^ values of 96, 95, and 94 percent were found, with SEPs of 5.39, 2.08, and 5.12 percent, respectively. The R^2^ values for frozen-thawed samples were 93, 95, and 82 percent, respectively, with SEP values of 7.11, 2.38, and 9.10 percent [166]. Ground beef adulterated with turkey was examined by UV–Vis (220–700 nm), NIR (942–2667 nm), and MIR (2701–3785, 4357–9920 nm) spectroscopy [167]. They prepared and examined 44 pure ground meat samples and 44 pure ground turke samples, as well as 154 ground beef adulterated with turkey meat in the range of 5–50 percent (w/w). Classification rates were obtained by NIR data (71.3%), while prediction of adulteration content was successful with R^2^ values of 98.3% [167]. On the other hand Rady and Adedeji (2018) studied the detection of adulteration of beef with chicken or pork and with vegetable protein using spectroscopic systems in the range of 400–1000 nm (visible/near-infrared or Vis–NIR) and 900–1700 nm (NIR) [166]. Adulterations with fractions of 1–50% were investigated. The classification rates of the first stage were 96% and 100% for pure/unadulterated and adulterated samples, respectively. In contrast, in the second stage, the classification rates were 69–100%. The result showed that the optimal models for predicting the adulteration content yielded a correlation coefficient, of 0.78–0.86, and the ratio of performance to deviation, RPD, of 1.19–1.98 [166].

Similarly, NIR was used to discriminate chicken, turkey, pork, beef, and lamb from homogenized meat samples [168]. The samples were divided into four groups white and red meat, poultry and pork, beef and lamb and poultry, chicken versus turkey [168]. Recently, there has been an increase in the number of published research papers dealing with the application of NIR spectroscopy for the study of food quality and authenticity. Since NIR spectroscopy is fast, relatively easy to use, and does not require chemicals. Therefore, this technique has the potential to be used more widely for food analysis and detection, Theliterature suggests that the use this method in conjunction with other detection techniques, such as dual-energy X-ray absorptiometry (DEXA), would allow estimation of not only the chemical components and quality characteristics of meat but also the composition of the entire carcass (fat, lean, and bone tissue content) [161].

#### Raman spectroscopy

The Raman spectroscopy method, uses monochromatic visible/near-infrared light from a laser to irradiate a food sample. The oscillation of molecules from the short-lived to the high-energy collision step and back to the original low energy state is called Rayleigh scattering. The method has some advantages such as the fact that no sample preparation is required and that it can be applied very quickly to food samples [169]. For meat and meat products, Raman spectroscopy can be used to identify meat types classify, oils and fats quantitatively predict composition, detect meat adulteration and authenticity with molecular based [166, 167, 170]. Recently, Raman spectroscopy was used to evaluate the authenticity of halal/ kosher meat for the detection and quantification of lard in butter. Adulteration leveln from 0 to 100% lard fat (w/w), were used and the results showed the high efficiency of Raman spectroscopy in quantifying lard fat in fatty butter samples, with a simple, robust, effective, inexpensive, and reliable application in butter quality control [171]. Raman spectroscopy has several advantages over NIR spectroscopy, including negligible water interference and ease of examination through glass or polymer packaging. In addition, hand-held Raman spectroscopy can be used as an excellent tool for on line or online analysis of adulteration analysis in industries such as agriculture and food. The range of fingerprint, which includes particularly distinct and characteristic spectral features of the investigated substances, is expected to be between 400 and 2000 cm^−1^ [171].

#### Laser-induced breakdown spectroscopy (LIBS)

LIBS is a fast and practical method for elemental analysis with qualitative and quantitative measurements of elemental composition with detection of atomic and molecular emission signals [172]. It involves the generation of atoms emitting characteristic light and recorded as LIBS spectrum. LIBS has been used to identify the pork and chicken species in beef samples [173]. For LIBS measurements, specific amounts of pork, beef, and poultry were purchased from various sources and processed into pellets. Identification was based on the differences in elemental composition of the various samples. The collected LIBS spectra were analyzed by chemometric methods, and the meat types were qualitatively separated by principal component analysis (PCA) with 83.37 yield.

#### Fluorescent light spectroscopy

Electromagnetic spectroscopy, which studies the fluorescence of a material, can explain a beam of light (usually visible light) emitted by electrons in the molecules of the material [174]. Fluorescence is the emission of light that occurs within nanoseconds of the absorption of light with a shorter wavelength. The difference between the exciting and emitted wavelengths, known as the Stokes shift, is the key property that makes fluorescence so strong. By completely filtering out the exciting light without blocking the emitted fluorescence, it is possible to see only the objects that fluoresce [175]. Fluorescence spectroscopy has been used in a variety of food detection applications, such as detecting adulteration in extra virgin olive oil [176]. However, with regard to halal authenticity, fluorescence spectroscopy has been used to identify pork adulteration in foods and beverages. In this experiment, different mixtures of foods (with and without pork) were examined and the light spectrum was measured using RSpec software. The results showed that fluorescence spectroscopy could distinguish pure pork, a mixture with pork, and without pork [174]. Munir et al. (2019) used the FTIR spectrum technique in combination with a partial least squares (PLS) method for the rapid detection of lard in sunflower, canola, coconut, olive, and mustard oils.

## Discussing and future perspectives

Given the increased concern among consumers regarding the authenticity of halal and/or kosher food products throughout the world, there is an urgent need to develop quick, reliable, and effective analytical detection methods. Therefore, the identification of specific food components with respect to halal/kosher authenticity of food products has been carried out using various methods and equipment. The use of these authenticity detection techniques was started with chromatographic and electrophoretic methods, but the mentioned methods are time consuming, require laboratory, are costly, and require skilled personnel. Genetic methods are specific/sensitive, but they require expensive laboratory equipment, and the methods are complex to perform. Immunological analyses are specific and simple, but the thresholds of the tests can be a problem.

In addition, the extraction of the adulteration markers requires much more time, chemicals, and equipment. Additionally, errors in extraction can lead to false results and difficulty in repeating the analysis. PCR-based techniques require a sample preparation step such as DNA extraction prior to analysis. DNA extraction of some foods has low yield and poor quality because DNA degrades during food processing at high temperatures. Calorimetric determination methods require few samples and provide accurate results, but the methods are not suitable for practical determination of industry expectations due to laboratory requirements. Mass spectrometry (MS) can be combined with HPLC and GC systems, but is associated with high cost, Chromatographic detection methods, especially those based one GC, are laborious and time-consuming. Another chromatography-based technique is Capillary Electrophoresis (CE), which is not sensitive to cooked meat. For the detection of fatty acid chain composition in meat products, NMR spectroscopy can be used as an alternative method instead of classical chromatographic methods. However, approaches based on MS currently offer higher sensitivity than NMR. Likewise, ELISA technique also has some disadvantages, such as the complexity of antibody preparation and the instability of antibodies due to their temperature sensitivity. All the mentioned analytical methods have been listed inTable 3 with their strengths and weaknesses.

Detection methods should be rapid, simple, easy to use, and repeatable in halal/kosher authenticity. Some protein or volatile based methods are affected by processing, storage, or environmental conditions. They are not suitable for halal/kosher food processing. The new methods can be readily used in the industrial process. In addition, detection methods for authentication and adulteration of a halal/kosher should be selective and sensitive with respect to the addition of pork, lard, or porcine gelatin. Similarly, methods for these purposes must be rapid, accurate, inexpensive, with minimal sample volumes, and no chemical waste. If the method is not much more complex or does not require sample preparation steps prior to analysis, it is an integrated production process or simple to use.

For this reason, fingerprint methods such as FTIR, NMR, NIR, Raman, LIBS, and fluorescence spectroscopy, especially portable devices, can be a very effective choice for halal/kosher detection of foods because the methods are fast, high throughput, sensitive, accurate, and non-destructive. Other advantages of the above methods include ease of sample preparation, no need reagents or solvents, and the use of qualified personnel. Last but not least, if the halal/kosher status of food products can be determined quickly, easily, and cost-effectively, consumers and producers will benefit from decreased product costs and selling prices.

**Table 1:** Summary of applications of chromatographic and conventional methods used for the detection of food adulteration and product quality, sorted by method, sample, issue, and publication year

Method	Sample	Issue	Research
Chromatographic methods			
HPLC	Processed food matrices	Detection of horse and pork	[36]
	Vegetable oils	Lard detection	[39]
	Fried chicken	Lard detection	[42]
GC- MS	Beef samples	Detection of pork adulteration	[48]
GC + SPME	Pig meats (cooked, frozen, raw)	Detect volatile compounds of pig meats	[47]
Conventional methods			
CE-SDS	Meat species (beef, turkey, pork, and turkey meats)	Identification of meat species	[58]
E-nose	Palm oil	Lard detection	[82]
	Mutton samples	Detection of pork	[65]
PCR	Beef or chicken raw meat mixtures	Detection of pork	[89]
	Meatballs	Detection of dog meat	[101]
	Cattle, goats, chickens, turkeys, and pigs	Differentiate the species	[131]
	Beef, pork, buffalo, quail*,* chicken*,* goat*, and* rabbit species	Differentiate the species	[73]
ELISA	Raw ground beef	Detection of raw pork	[118]
	Beef meat	Detection of low levels ( 0.01%) of pork adulteration	[119]
	Meat and food product	Quantification of pork	[132]
DSC			
	Ghee and butter	Adulteration of beef fat from cattle and buffalo with pig	[133]
		Detection of pigs and animal fat contaminated with pork fat	[134]
	Palm oil	Detection of lard	[127]
	Meatball	Detection of wild boar	[128]
	Canola oil	Adulteration with genuine lard, beef tallow, chicken fat	[130]

**Table 2:** Summary of applications of fingerprinting methods (FTIR, NMR, NIR, Raman, LIBS, and Fluorescence spectroscopy) for the detection of food adulteration and product quality, sorted by method, sample, issue, and publication year

Method	Sample	Issue	Research
FTIR	Vegetable oils	Detection of Lard adulteration	[23]
	animal fats (namely chicken, beef, and mutton fats)	Detection of Lard adulteration	[22]
	cod liver oil	Detection of Lard adulteration	[177]
	Beef meatballs	Quantification and classification of pork	[16]
	Chocolate	Detection of lard	[178]
	Meatball broth	Detection of Lard adulteration	[148]
	Sunflower, canola, coconut, olive, and mustard oils	Detection of Lard adulteration	[151]
NMR	canola oils	Detection of lard and beef tallow adulteration	[53]
	Butter	Detection lard amount	[25]
NIR	Veal sausage	Detection of pork adulteration	[163]
	Beef patties	Detection of pig, mutton, skim milk, or wheat flour in raw and cooked	[164]
	Beef samples	Detection of the adulteration of with chicken or pork and with vegetable protein	[166]
Raman	Butter	Detection of Lard adulteration	[171]
LIBS	Beef samples	Detection of pork and chicken	[173]
Fluorescence spectroscopy	Food and beverages	Detection of pork adulteration	[174]

**Table 3:** Strength and weakness sides of detection methods used to determine Halal/Kosher food products

Method	Strength	Weakness
Chromatographic methods		
HPLC	-Rapid and Precise -Automated process -High sensitivity -Quantitative recovery of samples	-Laborious -Slow, expensive, and invasive -No universal detector -Less separation efficiency than GC -High cost of columns
GC	-High sensitivity, resolution, and separation ability -Fast, low sample volume -Easy, automated, high precision and accuracy	-Used only for volatile and thermally stable compounds -Only the temperature of the column and oven can be control, but the mobile phase flow rate cannot be changed -Individual sample components cannot be recovered
Conventional methods		
MS	-High selectivity and sensitivity	-Needs to be combined with other techniques, such as gas chromatography (GC–MS)
CE	-Simple, automated, fast, low sample volume, low cost, separate both charged and non-charged molecules, high separation efficiency	-Sensitivity and resolution limits
EN	-Fast, high specificity for oxidized compound, low cost, very high sensitivity to virtually all gases, good precision	-High temperature operation -Sensitive only to oxygen containing compounds -Limited sensitivity to low molecular weight gases -High power consumption
PCR	-Real-time monitoring -Quantitative -Low possibility of contamination -High sensitivity	-Real-time monitoring -Quantitative -Low possibility of contamination -High sensitivity
ELISA	-Simple -High sensitivity and selectivity -High efficiency -No need for complex sample pre-treatment -Low reagents cost	-High probability of false positive or negative findings -The preparation of the antibodies is a sophisticated technique -The instability of the antibodies (needs refrigerated transport and storage)
DSC	-Quick analysis -Needs small sample amount -High sensitivity -High temperature -Stability of the materials	-Not always easy to interpret the results -Quantitative analysis is impossible -Cannot do both sensitivity and resolution analysis in one process -Very sensitive to any changes
Fingerprinting methods		
FTIR	-Simpler mechanical design -Sensitive, fast and easy -Relatively inexpensive -High throughput	- Semi-quantitative -False positives and false negatives numbers are possible -Many detected unknown -Not always easy to interpret the results
NMR	-Rapid -Nondestructive -High throughput	-Low sensitive than other analytical methods -NMR spectra are complex
NIR	-Simple -Low cost, high yield -Real time analysis -Measure multiple constituents simultaneously -Non-destructive -No chemical waste	-Calibration is less accurate -Small calibration sample sizes can lead to over confidence
Raman Spectroscopy	-Minimum sample preparation -Water does not interfere in the analysis -Fast -Easy examination via glass or polymer packaging -Noninvasive method	-Poor signal -The fluorescence is relatively strong and may overlap with the relatively weak Raman signal
LIBS	-A quick and simple method -Based multi-elemental analysis method -In-situ method (analyze solids, liquids and gases) -Minimum sample preparation -Quantitative and qualitative analysis	-High moisture or fat content can be problematic in obtaining high quality signal (drying or defatting process required) -Direct analysis of liquids might cause surface ripples and splashing
Fluorescence spectroscopy	-High sensitivity and specificity -Fast and rapid diagnosis ability	-Not all compounds fluoresce -Short lifespan of the phluorohphore -Susceptible to autophlorescence
